# Widespread sympatry in a species-rich clade of marine fishes (Carangoidei)

**DOI:** 10.1098/rspb.2023.0657

**Published:** 2023-11-01

**Authors:** Jessica R. Glass, Richard C. Harrington, Peter F. Cowman, Brant C. Faircloth, Thomas J. Near

**Affiliations:** ^1^ College of Fisheries and Ocean Sciences, University of Alaska Fairbanks, Fairbanks, AK 99775, USA; ^2^ South African Institute for Aquatic Biodiversity, Makhanda 6140, South Africa; ^3^ Department of Ecology and Evolutionary Biology, Yale University, New Haven, CT 06520, USA; ^4^ College of Science and Engineering, James Cook University, Townsville, Queensland 4811, Australia; ^5^ Biodiversity and Geosciences Program, Museum of Tropical Queensland, Queensland Museum, Townsville, Queensland 4810, Australia; ^6^ Department of Biological Sciences and Museum of Natural Science, Louisiana State University, Baton Rouge, LA 70803, USA; ^7^ Yale Peabody Museum of Natural History, Division of Vertebrate Zoology. New Haven, CT 06520, USA

**Keywords:** ultraconserved elements, marine fish speciation, age-range correlation, phylogenomics, carangiformes

## Abstract

A universal paradigm describing patterns of speciation across the tree of life has been debated for decades. In marine organisms, inferring patterns of speciation using contemporary and historical patterns of biogeography is challenging due to the deficiency of species-level phylogenies and information on species' distributions, as well as conflicting relationships between species’ dispersal, range size and co-occurrence. Most research on global patterns of marine fish speciation and biogeography has focused on coral reef or pelagic species. Carangoidei is an ecologically important clade of marine fishes that use coral reef and pelagic environments. We used sequence capture of 1314 ultraconserved elements (UCEs) from 154 taxa to generate a time-calibrated phylogeny of Carangoidei and its parent clade, Carangiformes. Age-range correlation analyses of the geographical distributions and divergence times of sister species pairs reveal widespread sympatry, with 73% of sister species pairs exhibiting sympatric geographical distributions, regardless of node age. Most species pairs coexist across large portions of their ranges. We also observe greater disparity in body length and maximum depth between sympatric relative to allopatric sister species. These and other ecological or behavioural attributes probably facilitate sympatry among the most closely related carangoids.

## 1. Introduction

For decades, biologists have debated whether there is a universal paradigm to explain patterns and processes of speciation in marine habitats [1–3]. From describing modes of speciation and mechanisms of dispersal [4,5], to characterizing latitudinal and longitudinal diversity gradients [6–8] and hypothesizing geographical origins of diversity [9–11], the rise of genetic methods and oceanographic modelling has upended traditional assumptions that vicariance leading to allopatry [12] is the default mechanism of speciation in the ocean [1,11]. Although biogeographic barriers have been shown to result in allopatric speciation in certain circumstances [1,13–15], in oceanic environments with fewer obvious geographical barriers to dispersal, other factors such as body size, pelagic larval duration and dispersal ability may be the prominent facilitators, rather than artefacts, of speciation [2,4,16–18].

To assess contemporary and historical patterns of marine speciation and biogeography, scientists have employed a variety of approaches [1,14,19–21]. One comparative method, age-range correlation, analyses the extent of range overlap between sister species pairs compared to the age of the phylogenetic node immediately subtending them as a proxy of species' age [22–26]. Age-range correlations can be examined across sister species pairs to look for associations between geographical patterns and relative node ages. Assuming an allopatric speciation model, random, independent changes in ranges over time should lead to greater sympatry at older nodes, whereas a sympatric speciation model should reflect greater range overlap in recently diverged sister species compared to more distantly related sister clades [23,24]. Peripatric speciation, caused when a population becomes isolated at the periphery of its ancestral distribution, can be assessed by examining range size evenness (range symmetry) between sister species. Peripatric speciation is thought to occur when the ranges of recently diverged sister species are highly asymmetrical due to one species having a smaller range on the edge of the larger ancestral range [22,27].

Few studies have applied analyses of age-range correlation and range symmetry in large and taxonomically inclusive lineages of vertebrates, particularly marine fishes [18,23,24,28–31]. The use of these approaches is hindered by a lack of comprehensive taxon sampling and limited availability of range data for many species. Existing age-range correlation studies on marine fishes have focused mainly on taxa occupying tropical coral reefs [1,6,11,14,32, cf. 33]. While these methods have challenges, such as distinguishing between sympatric speciation and secondary sympatry (i.e. allopatric speciation with subsequent range changes) [19,22,26,34], examining relationships between species ranges and node ages across large clades remains useful for understanding contemporary and historic biogeography in marine fishes. For pelagic and non-reef obligate species with high dispersal abilities, traditional models of allopatric and parapatric speciation that are thought to affect coral reef species [1,14] may be less important than sympatric speciation involving ecological divergence through habitat partitioning or reproductive timing [2,16,32]. Examining clade-level patterns of species ranges and integrating approaches such as age-range correlation with ecological data allow one to quantify the relationship between biogeography and speciation at larger taxonomic scales and assess potential drivers or outcomes of different speciation mechanisms (e.g. character displacement, competitive release).

**Figure 1.**
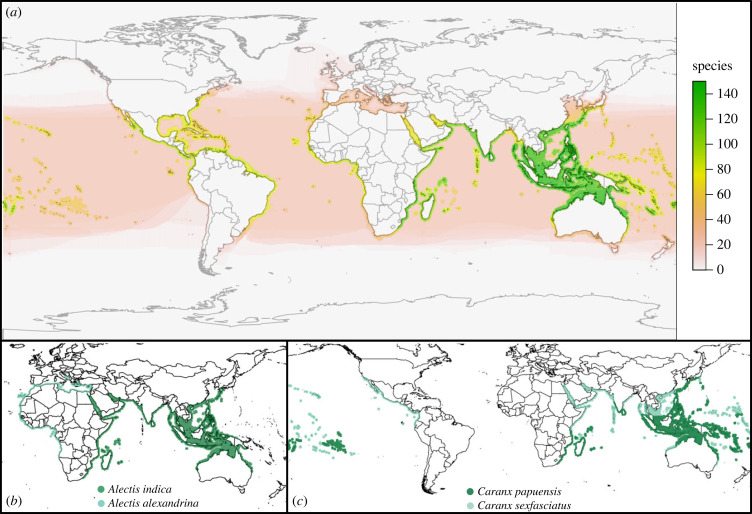
(*a*) Heatmap of Carangoidei species richness using species range extent of occurrence data from IUCN and probability of occurrence data from Aquamaps [46]. (*b*) Allopatric sister species pair, *Alectis indica* (dark green) and *Alectis alexandrina* (light green), exhibit no range overlap and low range symmetry. (*c*) Sympatric sister species pair, *Caranx sexfasciatus* (light green) and *Caranx papuensis* (dark green), exhibit high range overlap and high range symmetry. The dark green regions in (*c*) represent complete overlap between *C. sexfasciatus* and *C. papuensis*.

Here, we integrate ecological trait data and characterize contemporary patterns of biogeography in species belonging to a large clade of coastal-pelagic percomorph marine fishes, Carangoidei [35], which contains *Coryphaena* (dolphinfishes), Echeneidae (remoras), *Rachycentron canadum* (cobia) and Carangidae (trevallies). These fishes prefer habitats ranging from reef-associated with pelagic-neritic to brackish, although the group can broadly be classified as coastal-pelagic. Life-history characteristics of the carangoids frequently exclude them from studies on coral reef obligate fishes, as well as studies that focus on open-water pelagic species such as tunas, because they do not exhibit ecological traits characteristic of entirely one group. For example, some genera within Carangoidei (e.g. *Seriola*, *Caranx*, *Remora*) have high dispersal potential due to their large body size and association with drifting seaweed rafts, similar to pelagic fishes [17]. Yet many carangoid species also display restricted home ranges [36–39] – a trait more characteristic of reef fishes. Carangoids are assumed to have pelagic larval dispersal but the length of larval drifting and juvenile settlement patterns varies by species [40–42]. Moreover, the importance of pelagic larval duration on dispersal, range size and speciation rate for marine fishes remains disputed [18,43–45]. The regions of highest species richness of Carangoidei are the reef-abundant Indo-Australian Archipelago and Western Indian Ocean (figure 1*a*), making carangoids important for discussions on origins of tropical and sub-tropical fish biodiversity [6]. Carangoids are thus a key group for studying biogeographic patterns of fishes that span coral reefs and coastal habitats to the open ocean.

A comprehensive, time-calibrated phylogeny is highly desirable to study patterns of speciation. However, the monophyly and taxonomic composition of Carangoidei and the more inclusive lineage, Carangiformes, have been contentious since the first phylogenies of these groups were published in the late twentieth century [42,47,48]. For example, early morphological phylogenies suggested Carangoidei encompassed the Carangidae, Echeneidae, *Rachycentron canadum*, *Coryphaena*, *Nematistius pectoralis* (roosterfish) and *Mene maculata* (moonfish) [42,49,50], whereas molecular data consistently resolved Carangidae as paraphyletic, but only inclusive of *Rachycentron canadum*, *Coryphaena* and Echeneidae [35,51–53]. Moreover, molecular studies have disagreed on whether Carangiformes is paraphyletic [54,55] or monophyletic [35,56–58]. These prior studies have been limited by combinations of taxonomic or locus sampling [51,53,56] or insufficient fossil calibrations [52]. Here, we perform a comprehensive phylogenomic analysis using a dataset of more than 955 ultraconserved element (UCE) loci [59] collected from 80% of the recognized species of Carangoidei. We combine this phylogenetic framework with data on geographical distributions, depth distributions and body size to address patterns of allopatry and sympatry in Carangoidei and examine phylogenetic signal in traits thought to influence speciation.

## 2. Material and methods

### (a) Specimen sampling, genomic library construction and DNA sequencing

We obtained tissues for 154 species including nine outgroup species of Carangiformes through field collection and museum loans (electronic supplementary material, table S1). We prepared dual-indexed libraries [60] for targeted enrichment using the HyperPrep Kit (KAPA Biosystems, Wilmington, MA) following the manufacturer's protocols (electronic supplementary material). We used a probe set targeting 1314 UCE loci informative for phylogenetic analyses of Carangiformes and other acanthomorph fishes across evolutionary time scales [59]. We followed the methods of Ghezelayagh and Harrington [61]; see detailed protocol in electronic supplementary material. UCE sequence data were processed prior to phylogenetic analyses with *phyluce* v1.6 [62], which we used to construct alignments of individual UCE loci and perform edge trimming. We generated two data matrices for phylogenetic analyses to compare tree topologies with different amounts of missing data: one where 75% of taxa (115 out of 154) were present in each alignment and one where 95% of taxa (146 out of 154) were present.

### (b) Phylogenetic and relaxed molecular clock analyses

We implemented the UCE-specific Sliding Window Site Characteristics approach with site entropy (SWSC-EN) to identify UCE core and flanking regions at each locus [63]. We used these results as input for PartitionFinder v2 [64] to determine the optimal number of partitions for loci in the 75% complete and 95% complete matrices. We inferred a partitioned maximum-likelihood (ML) phylogeny using IQ-TREE [65] and implemented the ultrafast bootstrap approximation approach using 1000 bootstrap replicates and a relaxed hierarchical clustering algorithm (rcluster) that included the top 10% partition merging schemes [66]. We rooted the tree with the myctophid *Ceratoscopelus warmingii*.

To account for stochasticity in the evolutionary history among individual UCE loci, we also performed a coalescent-based analysis using loci from the 75% complete matrix. We first generated individual locus trees in MrBayes v3.2.6 (see electronic supplementary material) [67]. We inferred a species tree from these locus trees using ASTRAL-II v5.6.2 with the default parameters [68]. Given the similar topologies generated from the 75% complete concatenated dataset compared to the coalescent-based tree, we used the ML phylogeny as a fixed topology to estimate divergence times of carangiform lineages by implementing a relaxed molecular clock approach in BEAST v1.10.4 [69]. Because BEAST has computational limitations when analysing hundreds of loci simultaneously, we performed replicate analyses using different combinations of loci, *sensu* Harrington *et al*. [56] and Branstetter *et al*. [70]. We included nine fossil calibration points from Harrington *et al*. [56] that spanned the Carangiformes clade and assigned the same lognormal prior distributions to incorporate age priors for select nodes (electronic supplementary material).

### (c) Biogeographic and trait analyses

We obtained range data for 125 species from the IUCN Red List database, which consists of expert-validated range maps depicting the known ‘extent of occurrence’ of each species in the form of spatial polygons [71]. We obtained ranges for species missing from the IUCN database (*n* = 25) from Aquamaps, a database of species range predictions that uses a combination of occurrence data and species range modelling to assign relative probabilities of occurrence for each point coordinate [46]. Species for which no range data were available (*n* = 4) were trimmed from the time-calibrated phylogeny prior to biogeographic analyses. We also trimmed the time-calibrated phylogeny to only include carangoid taxa (Carangidae, Echeneidae, Coryphaenidae and Rachycentridae). The final dataset contained 123 species (electronic supplementary material, able S1).

To assess relationships between morphological traits, ecological traits and biogeography, we compiled trait data on maximum body length and maximum depth in the water column for the 125 carangoid species from the IUCN database [71]. If IUCN data were missing, we used FishBase [72]. We also compiled data on habitat class (reef or non-reef-associated) and diet (piscivorous or non-piscivorous) from a prior study [73] and the IUCN database [71].

We extracted divergence time estimates for each node of the time-calibrated phylogeny and conducted pairwise comparisons of range overlap and range symmetry [26]. We defined range overlap as the area occupied by a given species pair divided by the area of the species with a smaller range [26]. This produced an index ranging from 0 to 1, with 0 indicating no overlap (figure 1*b*) and 1 indicating complete overlap (figure 1*c*). Complete overlap meant both species co-occur throughout their entire respective ranges or that the range of one species is entirely encompassed by the other. To incorporate possible errors in points of occurrence, we classified species as allopatric if the range overlap index was less than 0.05 and sympatric if the range overlap index was greater than or equal to 0.05. We performed these analyses between all species in the phylogeny and extracted sister species pairs for additional analyses. A sister species pair was defined as two species sharing a unique common ancestor, i.e. an ancestor not shared with any other taxa in our phylogeny. We analysed 41 distinct sister species pairs, of which at least 32 we believe to be direct sister species (reciprocally monophyletic). The remaining nine pairs were uncertain due to unsampled species which may represent a closer relative to one of the species in those pairs. We hereon refer to sister species pairs inclusive of those believed to be direct sister species and those that exclusively share a single ancestor based on our phylogenetic sampling. To test for peripatry, we calculated range symmetry for each sister species pair, defined as the smaller of the two species' ranges divided by the sum of both species’ ranges [22]. The range symmetry metric falls between 0 and 0.5, where 0.5 indicates that both species have equal-sized ranges.

### (d) Phylogenetic signal in Carangoidei

To examine the ecological and physical similarity of closely related species in Carangoidei, we tested three continuous traits (maximum body length, maximum water column depth and range size) and two discrete traits (habitat and piscivory) for phylogenetic signal, defined as the tendency for related species to resemble each other more than they resemble species drawn at random from the tree [74,75]. For continuous traits, we tested for phylogenetic signal using Blomberg's *K* [75]. We tested for phylogenetic signal in the discrete traits—habitat (reef or non-reef) and piscivory (piscivorous or non-piscivorous)—using Fritz's *D* [76] (electronic supplementary material).

We tested for phylogenetic signal of range overlap and range symmetry in Carangoidei using multiple matrix regression by means of a partial Mantel test with 1000 phylogenetically informed permutations in ‘phytools’ in R [77,78]. Although the Mantel test has been criticized for its low power, it is a suitable option for testing phylogenetic signal in data that are inherently pairwise contrasts, such as measures of range overlap and symmetry [79].

We used linear regression to examine the relationships between divergence times for 41 sister species pairs, range overlap, and range symmetry because these metrics are hypothesized to be informative about speciation mechanisms [22,26,29]. We also used Welch's *t*-tests to statistically examine the association of allopatry and sympatry with ecological trait differences. For each sister species pair, we calculated trait contrasts: the differences in body length and maximum water column depth between sister species. We chose water depth as a proxy for species' utilization of the water column. Limited availability of data on minimum water column depth prohibited us from calculating depth distributions across Carangoidei. We recognized this limitation and used the most comprehensive depth datasets available for the clade. We also analysed differences in body length and water column depth for sympatric sister species categorized by habitat type, i.e. whether both sister species occupied the same or different habitat type (reef or non-reef). We excluded one sister pair, *Trachinotus mookalee* (Indian pompano) and *Trachinotus anak* (oyster pompano), because no maximum depth data were available for *T. mookalee* [71,72].

## 3. Results

### (a) Phylogenomic analyses and divergence times

We collected sequence data from an average of 958 loci for 154 individuals. Following alignment trimming, mean locus length was 972 bp (range: 319–1570 bp) and each locus contained a mean of 270.6 parsimony informative sites. The 75% complete alignment included 986 loci and the 95% complete alignment included 371 loci. PartitionFinder produced 143 partitions for the 75% complete matrix and 94 partitions for the 95% complete matrix. IQ-TREE (electronic supplementary material, figures S1 and S2) and ASTRAL (electronic supplementary material, figure S3) inferred similar trees with high ultrafast bootstrap support and local posterior probabilities, respectively (electronic supplementary material, figures S4 and S5). We observe similar topologies generated from the 75% complete concatenated dataset compared to the coalescent-based tree (electronic supplementary material, figure S4) and low Robinson-Foulds values between the trees (electronic supplementary material, table S3).

**Figure 2.**
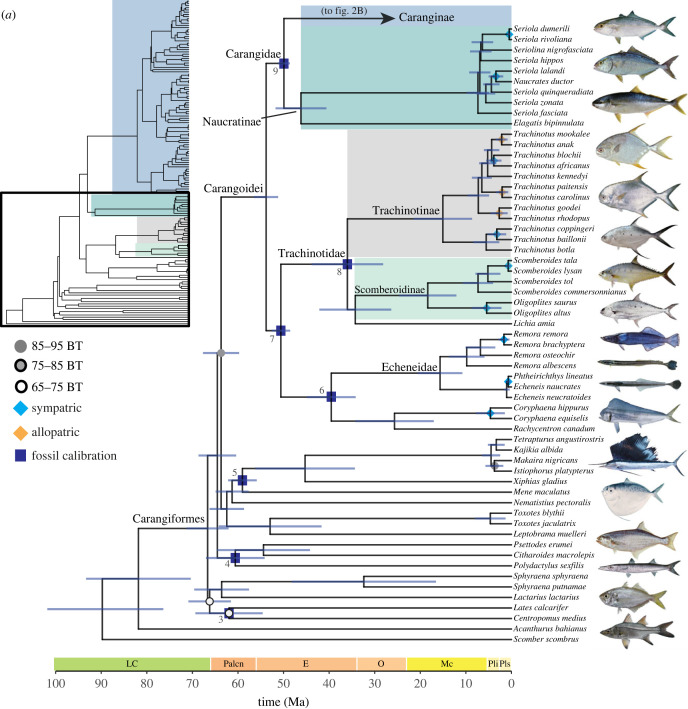

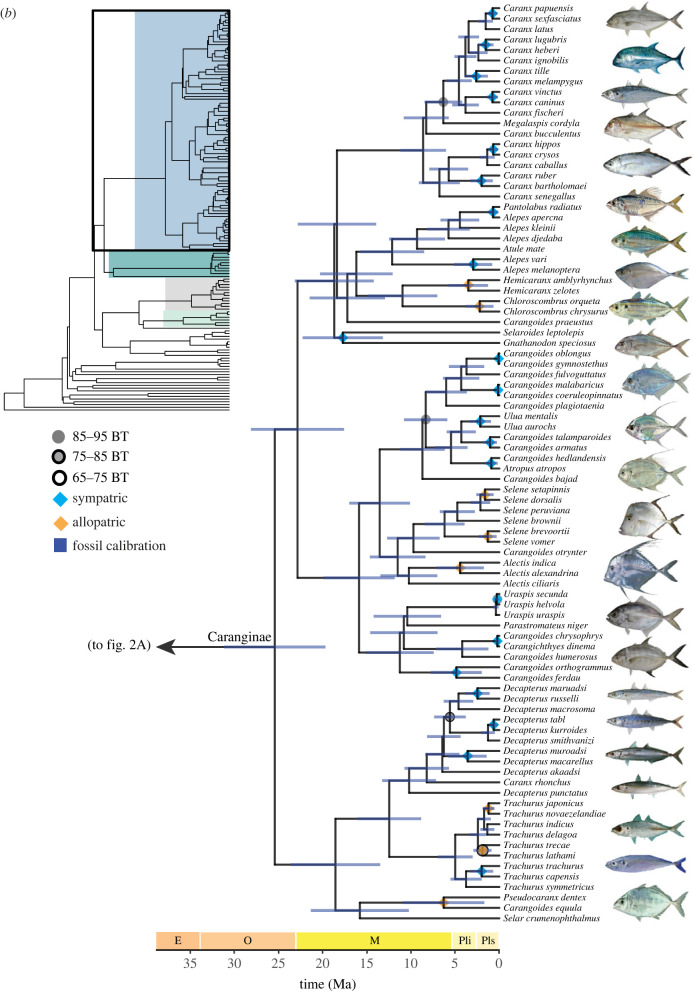
Time-calibrated phylogeny of 145 species of Carangiformes and two outgroup species generated by BEAST using a guide tree from the 75% complete matrix constructed in IQ-TREE and nine fossil calibration points. Blue bars indicate 95% posterior probability densities (HPD) around point estimates. Nodes represent median ages from a maximum clade-credibility tree. Ultrafast bootstrap support values (BT) are indicated as circles on each node. No circle indicates 95–100% BT support. Dark blue rectangles indicate nodes calibrated with priors based on fossil data. Diamond node labels indicate sympatric (light blue) and allopatric (black) carangoid sister species pairs. One sister pair (*Trachurus indicus* and *T. delagoa*) are unlabeled due to missing range data. Fish image sources are in electronic supplementary material, table S5.

Most nodes in the ML tree generated from the 75% complete matrix are strongly supported, with 90% having ultrafast bootstrap support of 100 (figure 2). Our phylogenomic analyses suggest four distinct lineages within carangiform fishes: (1) a clade containing *Lates calcarifer* (barramundi), Centropomidae, *Lactarius lactarius* and *Sphyraena*; (2) a clade containing Polynemidae and Pleuronectoidei (flatfishes); (3) a clade containing *Leptobrama*, *Toxotes*, *Nematistius pectoralis*, *Mene maculata* (moonfish), *Xiphias gladius* and Istiophoridae; and (4) a clade containing Echeneidae, *Rachycentron canadum* and *Coryphaena* nested in a paraphyletic Carangidae (figure 2*a*; electronic supplementary material, figures S1–S3).

Within Carangoidei, our phylogenetic hypotheses resolve two large subclades. The first consists of the echenoids (Echeneidae, *Rachycentron canadum* and *Coryphaena*). This clade is sister to the recently elevated Trachinotidae that includes *Trachinotus* (pompanos), *Lichia amia* (leerfish) and Scomberoidinae (*Oligoplites* [leatherjackets] and *Scomberoides* [queenfishes]) [80]. *Lichia amia* was previously classified with *Trachinotus* in Trachinotini [42,49]; however, we resolve *L. amia* as the sister lineage of Scomberoidinae with strong support (figure 2; electronic supplementary material, figures S1–S3). We delimit the second major subclade within Carangoidei as Carangidae, inclusive of Naucratinae and Caranginae, which contain numerous paraphyletic genera (figure 2). In *Alepes*, *Decapterus*, *Seriola*, and *Caranx,* one or two species classified in other genera resolve within these clades (figure 2; electronic supplementary material, figures S1–S3). *Carangoides* is polyphyletic, with species distributed across nine clades (figure 2*b*; electronic supplementary material, figures S1–S3). Our phylogenetic hypotheses are broadly congruent with recent molecular analyses focusing on Carangoidei that contain dense taxonomic sampling [52,53,56].

Species relationships within Carangiformes are largely consistent across the different methods of analysis (IQ-TREE, ASTRAL) and matrix composition (75% or 95% complete). Differences in phylogenetic relationships between the 75% ML topology and ASTRAL coalescent-based tree involve the phylogenetic placement of *Centropomus medius*, *Seriola nigrofasciata*, *Decapterus macarellus*, *D. akaadsi, Caranx crysos* and *C. caballus*, as well as *Parastromateus niger* (electronic supplementary material, figure S4). Maximum likelihood phylogenies inferred using the 75% and 95% matrices differ in the resolution of *Trachurus trecae*, *Decapterus akaadsi*, *Uraspis uraspis* and *Carangoides bajad* (electronic supplementary material, figure S5).

Using relaxed-clock molecular dating analyses, we generated similar estimates of divergence times and overlapping 95% highest posterior densities (HPD) across four random subsets of 25 UCE loci (figure 2; electronic supplementary material, figure S6). These analyses estimate the age of the most recent common ancestor (MRCA) of Carangiformes as 66.66 Ma (95% HPD: 62.43–71.59 Ma) and of Carangoidei as 53.82 Ma (95% HPD: 51.50–56.77 Ma; figure 2). These divergence times are younger than a previous study on Carangoidei [52] but fall within the 95% confidence intervals of other phylogenetic studies that estimate the ages of Carangiformes and Carangoidei [56–59].

### (b) Phylogenetic signal

Tests of continuous trait variables within Carangoidei using Blomberg's *K* suggest there is phylogenetic signal for body length (*K* = 0.123, *p* = 0.001), water column depth (*K* = 0.090, *p* = 0.006) and range size (*K* = 0.307, *p* = 0.001), as *K* values lower than one imply that variance is less than expected by a Brownian process. Phylogenetic least squares regression with an OU error model suggests a correlation between maximum body length and maximum water column depth (*t* = 2.593, *p* = 0.011), but not between maximum body length and geographical range size (*t* = 1.004, *p* = 0.317).

We calculated Fritz's *D* to examine phylogenetic signal in discrete variables (reef habitat and piscivory) and compared observed *D* values to simulated sums of expected character changes under Brownian motion and random models. The test for reef habitat (*D* = 0.663) suggests a departure from Brownian motion expectations (*p*[*D* > 0] < 0.001) but more phylogenetic signal than expected from a random distribution of habitat traits across the phylogeny (*p*[*D* < 1] < 0.001). The test of Fritz's *D* for diet (piscivory or non-piscivory; *D* = 0.069) suggests the evolution of diet resembles a Brownian process (*p*[*D* > 0] = 0.365; *p*[*D* < 1] < 0.001). Mantel tests of contrast variables reveal a correlation between phylogeny and overlap of geographical ranges (*R*^2^ = 0.020, *p* = 0.009), as well as phylogeny and geographical range size symmetry (*R*^2^ = 0.033, *p* = 0.001).

### (c) Sister species analyses

**Figure 3.**
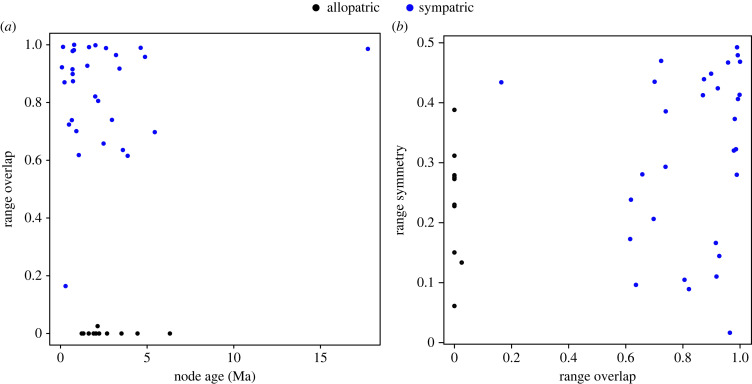
Range overlap as a function of node age (*a*) and range symmetry as a function of overlap (*b*) for 41 sister species pairs within Carangoidei.

**Figure 4.**
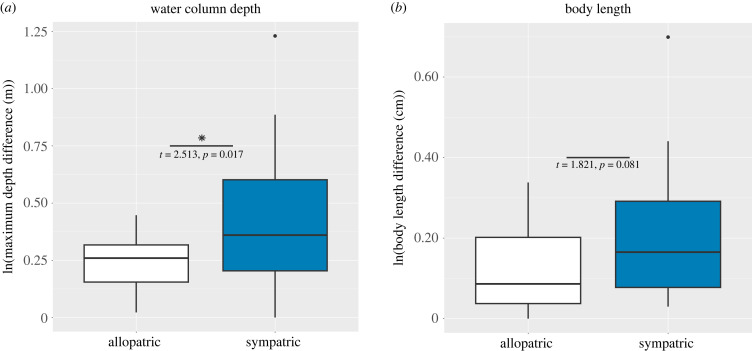
Contrasts between allopatric and sympatric sister species pairs for maximum water column depth (*a*) and maximum body length (*b*). Results of Welch's *t-*tests are presented to show significance between allopatric and sympatric sister species pairs.

Among the 41 resolved carangoid sister species pairs, 30 (73%) are sympatric (range overlap > 0.05) and 11 (27%) are allopatric (figure 2). All allopatric sister pairs have range overlap values of zero except *Trachinotus anak* and *T. mookalee*, with a range overlap value of 0.02. All sympatric species pairs have range overlap values > 0.6 except *Uraspis helvola* and *U. secunda*, whose range overlap is 0.16 (figure 3*a*). The node ages of sympatric sister species pairs range from 0.08–17.74 Ma (median: 1.65 Ma), while the node ages of allopatric pairs range from 1.23–6.31 Ma (median: 2.14 Ma). We find no effect of node age on range overlap (*r* = 2.25 × 10^−4^, *p* = 0.926; figure 3*a*) or geographical range size symmetry (*r* = 0.013, *p* = 0.480), nor is there a correlation between range overlap and range size symmetry (*r* = 0.072, *p* = 0.091; figure 3*b*). Median range size symmetry is 0.273 for allopatric species pairs and 0.348 for sympatric species pairs. Notably, there are greater differences in maximum water depth between sister species in sympatry versus those in allopatry (*t* = 2.513, *p* = 0.017; figure 4*a*). We also observe greater differences in maximum body length between sympatric sister species pairs compared to allopatric pairs (figure 4*b*), though these are not significant (*t* = 1.821, *p* = 0.081).

Most allopatric sister species pairs are comprised non-reef associated species. Out of 11 allopatric sister pairs, 73% (*n* = 8 pairs) contain two non-reef-associated species (electronic supplementary material, table S4). We also find greater differences in maximum water depth (*t* = 2.173, *p* = 0.034) between sympatric sister species pairs that occupy the same habitat (e.g. both occupy reef or non-reef habitats) compared to sympatric pairs that occupy different habitats (electronic supplementary material, figure S7).

## 4. Discussion

### (a) Carangiform phylogeny and timing of diversification

With a dataset averaging 958 UCE loci and representing 80% of the known species diversity within Carangoidei, we provide phylogenomic resolution for the relationships within this clade. The molecular and phylogenomic perspective on carangoid relationships is notable in the consistent paraphyly of the traditional delimitation of Carangidae when excluding the echenoids; thus, we confirm a newly revised classification for the carangoid subclades Carangidae and Trachinotidae [80]*.* Our resolution of subclades within Carangiformes is concordant with previous analyses using UCEs, with the exception of the relationships of Latidae, Centropomidae and *Sphyraena* [35,56]; these different phylogenetic relationships of early diverging carangiform lineages reflect ongoing challenges using molecular data to resolve taxonomic relationships due to short internal branches [81].

Our estimates of node ages suggest the origin of Carangoidei was approximately 53 Ma during the early Eocene (electronic supplementary material, figure S6). Estimates from UCE data for the age of Carangoidei are much younger than a previous study which suggested a Late Cretaceous origin; this is probably due to that study's fossil calibrations, which have older age estimates within Carangoidei [52]. Due to several identical fossil calibration points within Carangiformes shared across studies, our age estimates are similar to phylogenomic analyses using UCEs [56,59] and exons from protein coding genes [57]. Our results suggest most of the species level diversification occurred during the last 10 million years, during the late Miocene (approx. 11.63–5.33 Ma). The late Miocene was a period of warmer global climate and expanding coral reef habitats, which is congruent with the observed diversification of other tropical and sub-tropical coral reef fish lineages with diversity centered in the Indo-Pacific Archipelago region [6,9].

### (b) Patterns of carangoid sympatry and allopatry

While sympatry of sister species pairs is ubiquitous across Carangoidei, regardless of node age, 27% of sister species pairs were allopatric. Most cases of allopatry (64%) were likely caused by vicariance, specifically the Isthmus of Panama. Seven out of eleven allopatric pairs have divergence times younger than 5 Ma and presently exhibit parallel range patterns, where one species occupies the eastern Pacific (e.g. *Selene brevoortii* [Mexican lookdown]) and its sister species inhabits the western Atlantic (e.g. *Selene vomer* [lookdown]; electronic supplementary material, figure S8A). The other cases of allopatry are potentially maintained by the cold-water barrier formed by the Benguela and Agulhas currents off the southern coast of South Africa separating the Atlantic and Indian Oceans (*Alectis indica* [diamond trevally] and *A. alexandrina* [African threadfish]), or open-ocean barriers in the Atlantic (e.g. *Selene setapinnis* [Atlantic moonfish] and *S. dorsalis* [African moonfish]; electronic supplementary material, figure S8B) and Indo-Pacific (*Trachinotus mookalee* and *T. anak*; *Trachurus japonicus* [Japanese horse mackerel] and *T. novaezelandiae* [yellowtail horse mackerel]). Isolating barriers are unknown for the oldest (approx. 6.3 Ma) diverging allopatric sister pair, *Pseudocaranx dentex* (white trevally) and *Carangoides equula* (whitefin trevally), which are found throughout the Atlantic and Indo-Pacific, respectively.

To our knowledge, only one study has demonstrated such widespread sympatry (76%) in a large lineage of marine fishes, Myctophidae [82], although high degrees of sympatry have been demonstrated in some genera of coral reef fishes – for example, over 80% of sister species pairs in *Pomacanthus* (angelfishes; *n* = 13 spp.) [83] and *Haemulon* (grunts; *n* = 21 spp.) [84]. Most comparable analyses of marine fishes found a higher prevalence of allopatry between sister species, from 62% in New World haemulid fishes (*n* = 42 spp.) [31] to 64% in parrotfishes (*N* = 61 spp.) [85] to 88% in *Halichoeres* (wrasses; *n* = 24 spp.) [86]. In *Holocanthus* (angelfishes; *n* = 7 spp.), one sister pair is sympatric while the other species are allopatric [87].

Our age-range correlation analysis revealed patterns that were not clearly consistent with a single geographical mechanism of speciation (e.g. sympatry, allopatry, peripatry), potentially due to range shifts in this clade over time [19]. While we cannot rule out sympatric speciation due to lack of appropriate data, we follow long-held assumptions and empirical evidence that allopatric speciation is the predominant mechanism driving diversification [88,89]. As such, the clade-wide pattern we observed may suggest secondary sympatry occurring after allopatric speciation. Moreover, the patterns we observe in range overlap and range symmetry imply close relatives in Carangoidei coexist and maintain sympatry across large portions of their ranges. The greater divergence in body size and water column depth in sympatric sister pairs compared to allopatric pairs may be prezygotic isolation mechanisms that reduce interspecific competition (e.g. character displacement), facilitating secondary sympatry among closely related species [90]. Similar examples of transitions from allopatry to secondary sympatry are sparse but have been observed in birds [30,91,92] and coral reef fishes [25,93]. Even under an assumed model of allopatric speciation, recent evidence suggests diverging and recently diverged lineages of birds, mammals and amphibians evolve under similar macro-selective pressures, contradicting long-standing ideas that divergent, allopatric adaptation initiates the earliest stages of speciation [33]. Reef fishes and marine cetaceans exhibit higher transition rates to sympatry than birds and other vertebrate lineages [93]. Although node age is a significant predictor of transition from allopatry to sympatry in terrestrial organisms, the probability of sympatry is independent of node age in coral reef fishes and cetaceans due to frequent, fast transitions between allopatric and sympatric states [93]. These fast transitions are attributed to higher intrinsic dispersal abilities in lineages of marine organisms compared to terrestrial vertebrates [93] even though dispersal ability—including pelagic larval duration—has a nuanced correlation with range size [18,44].

### (c) Ecological signature of secondary sympatry in carangoid fishes

We observe higher divergence in maximum water depth and body size in sympatric sister species pairs, suggesting ecological factors facilitate sympatry among the most closely related species of carangoids. Water depth differences between sympatric sister species have also been documented in New World *Halichoeres* fishes [86], and sympatric sister pairs of parrotfish exhibit greater differences in body size, morphology, habitat type and colour patterns [85]. Body size and water column depth are reflective of resource use, with the former being a strong correlate of prey consumption [94] and the latter indicative of habitat niche partitioning [95,96]. The trait differences we examined in Carangoidei may be the result of character displacement, which is represented by the divergence of character traits in two or more lineages occurring in sympatry [90,97–100], but at present, character displacement is difficult to prove due to the lack of detailed ecological trait data across carangoid species' ranges. Given that body size and maximum depth in the water column are positively correlated [101], it is unclear if divergence in body size is driving divergence in water depth distribution. Further research on these and other traits is warranted, particularly to compare sister species’ traits between areas of overlap versus non-overlap.

The displacement of ecological and behavioural characters, in part by minimizing competition, is hypothesized to facilitate sympatry between closely related species [97,99]. In coral reef environments, habitat complexity may influence character displacement in reef fishes, be it divergence in mate recognition [102], trophic partitioning [103], reef preference [85], or territoriality [104]. Yet, since fewer than half of carangoid species (45%) are classified as reef-associated, carangoid niche partitioning might be shaped by different factors than those affecting coral reef fishes. Most (80%) allopatric carangoid sister pairs are non-reef-associated, while 80% of sympatric sister species contain at least one reef-associated species. A previous analysis on carangoid body shape and ecological traits found that shifts from reef to non-reef environments increased rates of morphological diversification, implying that non-reef environments influenced morphological changes more than reef environments [73]. Although the authors did not find an effect of habitat type on rates of phylogenetic lineage diversification, their lineage diversification rates may have been skewed by their age estimates of Carangoidei [52], which are substantially older than our age estimates and those of other phylogenomic studies [56,57]. Our results suggest habitat and diet resemble a Brownian motion model of trait evolution, but we did not test for the effects of trait evolution on rates of diversification. Ecological partitioning among closely related species occupying non-reef environments might be one reason why carangoids exhibit such high disparity in body shape and body size relative to other percomorphs [48,105]. Despite this variation in body shape and size, we still observe phylogenetic signal in body length, which corroborates previous morphological work suggesting similarity in the evolution of carangoid body types among major subclades [51].

Our tests of phylogenetic covariance suggest that the evolution of certain morphological and ecological traits has been conserved during carangoid lineage diversification. Notably, although we observe weak but significant phylogenetic signal in body length and water column depth in Carangoidei, the prevalence of sympatry coincides with evidence of morphological and environmental niche-partitioning in body size and depth in the water column between sister taxa. Our results highlight the benefits of performing sister species analyses, not only because such analyses pose less risk of overestimating divergence times due to extinction events [24], but also because independent replicates are less likely to be phylogenetically confounded [106] and may reveal trait divergences that are masked by analyses of phylogenetic signal across the entire clade. Additional studies examining the mechanistic processes underlying speciation in Carangoidei, including mate selection, reproductive timing, and mechanisms of dispersal at the species-level will shed further light on the drivers of speciation in this unique clade of fishes.

## Data Availability

All data underlying the analyses of this work are available on Dryad (https://doi.org/10.5061/dryad.37pvmcvm1) [107]. Sequence data generated for this manuscript are archived as raw reads in the NCBI Sequence Repository (SRA) under NCBI BioProjects PRJNA1028788, PRJNA758064 and PRJNA341709. Supplementary material is available online [108].

## References

[1] Rocha LA, Bowen BW. 2008 Speciation in coral-reef fishes. J. Fish Biol. **72**, 1101-1121.

[2] Palumbi SR. 1994 Genetic divergence, reproductive isolation, and marine speciation. Annu. Rev. Ecol. Syst. **25**, 547-572.

[3] Jordan DS. 1905 The origin of species through isolation. Science **22**, 545-562. 17832412 10.1126/science.22.566.545

[4] Luiz OJ, Madin JS, Robertson DR, Rocha LA, Wirtz P, Floeter SR. 2012 Ecological traits influencing range expansion across large oceanic dispersal barriers: insights from tropical Atlantic reef fishes. Proc. R. Soc. B **279**, 1033-1040. (10.1098/rspb.2011.1525) PMC3259933 21920979

[5] Butlin RK, Galindo J, Grahame JW. 2008 Sympatric, parapatric or allopatric: the most important way to classify speciation? Phil. Trans. R. Soc. B **363**, 2997-3007. (10.1098/rstb.2008.0076) 18522915 PMC2607313

[6] Cowman PF. 2014 Historical factors that have shaped the evolution of tropical reef fishes: a review of phylogenies, biogeography, and remaining questions. Front. Genet. **5**, 394. (10.3389/fgene.2014.00394) 25431581 PMC4230204

[7] Hodge JR, van Herwerden L, Bellwood DR. 2014 Temporal evolution of coral reef fishes: global patterns and disparity in isolated locations. J. Biogeogr. **41**, 2115-2127. (10.1111/jbi.12356)

[8] Siqueira AC, Oliveira-Santos LGR, Cowman PF, Floeter SR. 2016 Evolutionary processes underlying latitudinal differences in reef fish biodiversity. Glob. Ecol. Biogeogr. **25**, 1466-1476. (10.1111/geb.12506)

[9] Cowman PF, Bellwood DR. 2013 The historical biogeography of coral reef fishes: global patterns of origination and dispersal. J. Biogeogr. **40**, 209-224. (10.1111/jbi.12003)

[10] Briggs JC. 2003 Marine centres of origin as evolutionary engines. J. Biogeogr. **30**, 1-18.

[11] Bowen BW, Rocha LA, Toonen RJ, Karl SA. 2013 The origins of tropical marine biodiversity. Trends Ecol. Evol. **28**, 359-366. (10.1016/j.tree.2013.01.018) 23453048

[12] Mayr E. 1942 Systematics and the origin of species from the viewpoint of a zoologist. Boston, MA: Harvard University Press.

[13] DiBattista JD, Berumen ML, Gaither MR, Rocha LA, Eble JA, Choat JH, Craig MT, Skillings DJ, Bowen BW. 2013 After continents divide: comparative phylogeography of reef fishes from the Red Sea and Indian Ocean. J. Biogeogr. **40**, 1170-1181. (10.1111/jbi.12068)

[14] Hodge JR, Bellwood DR. 2016 The geography of speciation in coral reef fishes: the relative importance of biogeographical barriers in separating sister-species. J. Biogeogr. **43**, 1324-1335. (10.1111/jbi.12729)

[15] Lessios HA. 2008 The great American schism: divergence of marine organisms after the rise of the Central American Isthmus. Ann. Rev. Ecol. Evol. Syst. **39**, 63-91. (10.1146/annurev.ecolsys.38.091206.095815)

[16] Norris RD. 2000 Pelagic species diversity, biogeography, and evolution. Paleobiology **26**, 236-258. (10.1666/0094-8373(2000)26[236:PSDBAE]2.0.CO;2)

[17] Luiz OJ, Allen AP, Robertson DR, Floeter SR, Madin JS. 2015 Seafarers or castaways: ecological traits associated with rafting dispersal in tropical reef fishes. J. Biogeogr. **42**, 2323-2333. (10.1111/jbi.12574)

[18] Mora C, Treml EA, Roberts J, Crosby K, Roy D, Tittensor DP. 2012 High connectivity among habitats precludes the relationship between dispersal and range size in tropical reef fishes. Ecography **35**, 89-96. (10.1111/j.1600-0587.2011.06874.x)

[19] Losos JB, Glor RE. 2003 Phylogenetic comparative methods and the geography of speciation. Trends Ecol. Evol. **18**, 220-227. (10.1016/S0169-5347(03)00037-5)

[20] Knowlton N. 2003 Sibling species in the sea. Annu. Rev. Ecol. Syst. **24**, 189-216. (10.1146/annurev.es.24.110193.001201)

[21] Landis MJ, Matzke NJ, Moore BR, Huelsenbeck JP. 2013 Bayesian analysis of biogeography when the number of areas is large. Syst. Biol. **62**, 789-804. (10.1093/sysbio/syt040) 23736102 PMC4064008

[22] Barraclough TG, Vogler AP. 2000 Detecting the geographical pattern of speciation from species-level phylogenies. Am. Nat. **155**, 419-434. (10.1086/303332) 10753072

[23] Fitzpatrick B, Turelli M. 2006 The geography of mammalian speciation: mixed signals from phylogenies and range maps. Evolution **60**, 601-615. 16637504

[24] Hodge J, Bellwood DR. 2015 On the relationship between species age and geographical range in reef fishes: Are widespread species older than they seem? Glob. Ecol. Biogeogr. **24**, 495-505. (10.1111/geb.12264)

[25] Quenouille B, Hubert N, Bermingham E, Planes S. 2011 Speciation in tropical seas: allopatry followed by range change. Mol. Phylogenet. Evol. **58**, 546-552. (10.1016/j.ympev.2010.12.009) 21256237

[26] Chesser RT, Zink RM. 1994 Modes of speciation in birds: A test of Lynch's method. Evolution **48**, 490-497. (10.2307/2410107) 28568302

[27] Hodge JR, Read CI, van Herwerden L, Bellwood DR. 2012 The role of peripheral endemism in species diversification: evidence from the coral reef fish genus *Anampses* (Family: Labridae). Mol. Phylogenet. Evol. **62**, 653-663. (10.1016/j.ympev.2011.11.007) 22122942

[28] Wollenberg KC, Vieites DR, Glaw F, Vences M. 2011 Speciation in little: the role of range and body size in the diversification of Malagasy mantellid frogs. BMC Evol. Biol. **11**, 217. (10.1186/1471-2148-11-217) 21777445 PMC3199771

[29] Anacker BL, Strauss SY. 2014 The geography and ecology of plant speciation: range overlap and niche divergence in sister species. Proc. R. Soc. B **281**, 20132980. (10.1098/rspb.2013.2980) PMC3906944 24452025

[30] Phillimore AB, Orme CDL, Thomas GH, Blackburn TM, Bennett PM, Gaston KJ, Owens IPF. 2008 Sympatric speciation in birds is rare: insights from range data and simulations. Am. Nat. **171**, 646-657. (10.1086/587074) 18419572

[31] Tavera JJ, Wainwright PC. 2019 Geography of speciation affects rate of trait divergence in haemulid fishes. Proc. R. Soc. B **286**, 20182852. (10.1098/rspb.2018.2852) PMC6408603 30963939

[32] Rocha LA, Robertson DR, Roman J, Bowen BW. 2005 Ecological speciation in tropical reef fishes. Proc. R. Soc. B **272**, 573-579. (10.1098/2004.3005) PMC1564072 15817431

[33] Anderson SAS, Weir JT. 2022 The role of divergent ecological adaptation during allopatric speciation in vertebrates. Science **378**, 1214-1218. (10.1126/science.abo7719) 36520892

[34] Pontarp M, Ripa J, Lundberg P. 2015 The biogeography of adaptive radiations and the geographic overlap of sister species. Am. Nat. **186**, 565-581. (10.1086/683260) 26655771

[35] Girard MG, Davis MP, Smith WL. 2020 The phylogeny of carangiform fishes: morphological and genomic investigations of a new fish clade. Copeia **108**, 265. (10.1643/CI-19-320)

[36] Meyer CG, Holland KN, Papastamatiou YP. 2007 Seasonal and diel movements of giant trevally *Caranx ignobilis* at remote Hawaiian atolls: implications for the design of marine protected areas. Mar. Ecol. Prog. Ser. **333**, 13-25. (10.3354/meps333013)

[37] Afonso P, Fontes J, Holland KN, Santos RS. 2009 Multi-scale patterns of habitat use in a highly mobile reef fish, the white trevally *Pseudocaranx dentex*, and their implications for marine reserve design. Mar. Ecol. Prog. Ser. **381**, 273-286. (10.3354/meps07946)

[38] Ross SW, Lancaster JE. 2002 Movements and site fidelity of two juvenile fish species using surf zone nursery habitats along the southeastern North Carolina coast. Environ. Biol. Fishes **63**, 161-172. (10.1023/A:1014287917297)

[39] Fontes J, Schmiing M, Afonso P. 2014 Permanent aggregations of a pelagic predator at shallow seamounts. Mar. Biol. **161**, 1349-1360. (10.1007/s00227-014-2423-9)

[40] Sudekum AE, Parrish JD, Radtke RL, Ralston S. 1991 Life history and ecology of large jacks in undisturbed, shallow, oceanic communities. Fish. Bull. **89**, 493-513.

[41] Leis JM, Hay AC, Lockett MM, Chen JP, Fang LS. 2007 Ontogeny of swimming speed in larvae of pelagic-spawning, tropical, marine fishes. Mar. Ecol. Prog. Ser. **349**, 255-267. (10.3354/meps07107)

[42] Smith-Vaniz WF. 1984 Carangidae: Relationships. In Ontogeny and systematics of fishes (eds HG Moser, WJ Richards, DM Cohen, MP Fahay, AW Kendall Jr, SL Richardson), pp. 522-530. Lawrence, KS: American Society of Ichthyologists and Herpetologists.

[43] Lester SE, Ruttenberg BI. 2005 The relationship between pelagic larval duration and range size in tropical reef fishes: a synthetic analysis. Proc. R. Soc. B **272**, 585-591. (10.1098/rspb.2004.2985) PMC1564084 16007745

[44] Lester SE, Ruttenberg BI, Gaines SD, Kinlan BP. 2007 The relationship between dispersal ability and geographic range size. Ecol. Lett. **10**, 745-758. (10.1111/j.1461-0248.2007.01070.x) 17594430

[45] Weersing K, Toonen RJ. 2009 Population genetics, larval dispersal, and connectivity in marine systems. Mar. Ecol. Prog. Ser. **393**, 1-12. (10.3354/meps08287)

[46] Kaschner K, Kesner-Reyes K, Garilao C, Segschneider J, Rius-Barile J, Rees T, Froese R. 2019. AquaMaps: predicted range maps for aquatic species. Retrieved from https://www.aquamaps.org (accessed on 25 November 2018).

[47] Bannikov AF. 1986 On the taxonomy, composition and origin of the family Carangidae. Voprosy Ikhtiologii **6**, 833-889.

[48] Gushiken S. 1988 Phylogenetic relationships of the perciform genera of the family Carangidae. Jap. J. lchthyol. **34**, 443-461. (10.11369/jji1950.34.443)

[49] Nelson JS, Grande TC, Wilson MVH. 2016 Fishes of the world. Hoboken, NJ: Wiley.

[50] Smith-Vaniz WF. 1984 Carangidae. In FAO species identification sheets for fishery purposes: Western Indian Ocean fishing area 51. (eds W Fischer, G Bianchi), Rome: FAO.

[51] Reed DL, Carpenter KE, deGravelle MJ. 2002 Molecular systematics of the jacks (Perciformes: Carangidae) based on mitochondrial cytochrome b sequences using parsimony, likelihood, and Bayesian approaches. Mol. Phylogenet. Evol. **23**, 513-524. (10.1016/S1055-7903(02)00036-2) 12099802

[52] Santini F, Carnevale G. 2015 First multilocus and densely sampled timetree of trevallies, pompanos and allies (Carangoidei, Percomorpha) suggests a Cretaceous origin and Eocene radiation of a major clade of piscivores. Mol. Phylogenet. Evol. **83**, 33-39. (10.1016/j.ympev.2014.10.018) 25450104

[53] Damerau M, Freese M, Hanel R. 2017 Multi-gene phylogeny of jacks and pompanos (Carangidae), including placement of monotypic vadigo *Campogramma glaycos*. J. Fish Biol. **92**, 190-202. (10.1111/jfb.13509) 29193148

[54] Betancur-R R et al. 2013 The tree of life and a new classification of bony fishes. PLOS Curr. Tree Life **0732988**, 1-45. (http://currents.plos.org/treeoflife/article/the-tree-of-life-and-a-new-classification-of-bony-fishes/) 10.1371/currents.tol.53ba26640df0ccaee75bb165c8c26288 PMC3644299 23653398

[55] Betancur-R R, Li C, Munroe TA, Ballesteros JA, Ortí G. 2013 Addressing gene tree discordance and non-stationarity to resolve a multi-locus phylogeny of the flatfishes (Teleostei: Pleuronectiformes). Syst. Biol. **62**, 763-785. (10.1093/sysbio/syt039) 23749787

[56] Harrington RC, Faircloth BC, Eytan RI, Smith WL, Near TJ, Alfaro ME, Friedman M. 2016 Phylogenomic analysis of carangimorph fishes reveals flatfish asymmetry arose in a blink of the evolutionary eye. BMC Evol. Biol. **16**, 224. (10.1186/s12862-016-0786-x) 27769164 PMC5073739

[57] Hughes LC et al. 2018 Comprehensive phylogeny of ray-finned fishes (Actinopterygii) based on transcriptomic and genomic data. Proc. Natl Acad. Sci. USA, **115**, 6249-6254. (10.1073/pnas.1719358115) 29760103 PMC6004478

[58] Near TJ et al. 2013 Phylogeny and tempo of diversification in the superradiation of spiny-rayed fishes. Proc. Natl Acad. Sci. USA **110**, 12 738-12 743. (10.1073/pnas.1304661110) PMC3732986 23858462

[59] Alfaro ME, Faircloth BC, Harrington RC, Sorenson L, Friedman M, Thacker CE, Oliveros CH, Černý D, Near TJ. 2018 Explosive diversification of marine fishes at the Cretaceous–Palaeogene boundary. Nature Ecology & Evolution **2**, 688-696. (10.1038/s41559-018-0494-6) 29531346

[60] Glenn TC et al. 2019 Adapterama I: universal stubs and primers for 384 unique dual-indexed or 147456 combinatorially-indexed Illumina libraries (iTru & iNext). PeerJ **7**, e7755. (10.7717/peerj.7755) 31616586 PMC6791352

[61] Ghezelayagh A et al. 2022 Prolonged morphological expansion of spiny-rayed fishes following the end-Cretaceous. Nat. Ecol. Evol. **6**, 1211-1220. (10.1038/s41559-022-01801-3) 35835827

[62] Faircloth BC. 2016 PHYLUCE is a software package for the analysis of conserved genomic loci. Bioinformatics **32**, 786-788. (10.1093/bioinformatics/btv646) 26530724

[63] Tagliacollo VA, Lanfear R. 2018 Estimating improved partitioning schemes for ultraconserved elements. Mol. Biol. Evol. **35**, 1798-1811. (10.1093/molbev/msy069) 29659989 PMC5995204

[64] Lanfear R, Frandsen PB, Wright AM, Senfeld T, Calcott B. 2016 PartitionFinder 2: New methods for selecting partitioned models of evolution for molecular and morphological phylogenetic analyses. Mol. Biol. Evol. **34**, 772-773. (10.1093/molbev/msw260) 28013191

[65] Nguyen LT, Schmidt HA, Von Haeseler A, Minh BQ. 2015 IQ-TREE: A fast and effective stochastic algorithm for estimating maximum-likelihood phylogenies. Mol. Biol. Evol. **32**, 268-274. (10.1093/molbev/msu300) 25371430 PMC4271533

[66] Minh BQ, Nguyen MAT, Von Haeseler A. 2013 Ultrafast approximation for phylogenetic bootstrap. Mol. Biol. Evol. **30**, 1188-1195. (10.1093/molbev/mst024) 23418397 PMC3670741

[67] Ronquist F et al. 2012 MrBayes 3.2: Efficient Bayesian phylogenetic inference and model choice across a large model space. Syst. Biol. **61**, 539-542. (10.1093/sysbio/sys029) 22357727 PMC3329765

[68] Mirarab S, Warnow T. 2015 ASTRAL-II: Coalescent-based species tree estimation with many hundreds of taxa and thousands of genes. Bioinformatics **31**, i44-i52. (10.1093/bioinformatics/btv234) 26072508 PMC4765870

[69] Suchard MA, Lemey P, Baele G, Ayres DL, Drummond AJ, Rambaut A. 2018 Bayesian phylogenetic and phylodynamic data integration using BEAST 1.10. Virus Evol. **4**, 1-5. (10.1093/ve/vey016) PMC6007674 29942656

[70] Branstetter MG, Danforth BN, Pitts JP, Faircloth BC, Ward PS, Buffington ML, Gates MW, Kula RR, Brady SG. 2017 Phylogenomic insights into the evolution of stinging wasps and the origins of ants and bees. Current Biology **27**, 1019-1025. (10.1016/j.cub.2017.03.027) 28376325

[71] IUCN. 2018 *The IUCN Red List of Threatened Species*. Version 2018-1. See http://www.iucnredlist.org (accessed on 14 September 2018).

[72] Froese D, Pauly D. 2000 Fishbase 2000: concepts, design and data sources. Laguna, Philippines: ICLARM.

[73] Frédérich B, Marramà G, Carnevale G, Santini F. 2016 Non-reef environments impact the diversification of extant jacks, remoras and allies (Carangoidei, Percomorpha). Proc. R. Soc. B **283**, 20161556. (10.1098/rspb.2016.1556) PMC5124091 27807262

[74] Hillis DM, Huelsenbeck JP. 1992 Signal, noise, and reliability in molecular phylogenetic analyses. Journal of Heredity **83**, 189-195. (10.1093/oxfordjournals.jhered.a111190) 1624764

[75] Blomberg SP, Garland T, Ives AR. 2003 Testing for phylogenetic signal in comparative data: behavioral traits are more labile. Evolution **57**, 717-745. (10.1111/j.0014-3820.2003.tb00285.x) 12778543

[76] Fritz SA, Purvis A. 2010 Selectivity in mammalian extinction risk and threat types: a new measure of phylogenetic signal strength in binary traits. Conserv. Biol. **24**, 1042-1051. (10.1111/j.1523-1739.2010.01455.x) 20184650

[77] Revell LJ. 2012 phytools: An R package for phylogenetic comparative biology (and other things). Methods Ecol. Evol. **3**, 217-223. (10.1111/j.2041-210X.2011.00169.x)

[78] Mantel N. 1967 The detection of disease clustering and a generalized regression approach. Cancer Res. **1**, 209-220. (10.1038/214637b0) 6018555

[79] Harmon LJ, Glor RE. 2010 Poor statistical performance of the Mantel test in phylogenetic comparative analyses. Evolution **64**, 2173-2178. (10.1111/j.1558-5646.2010.00973.x) 20163450

[80] Near TJ, Thacker CE. 2023 Phylogenetic classification of living and fossil ray-finned fishes (Actinopterygii). Bull. Peabody Museum Nat. Hist. **65**, 340–357. (10.5281/ZENODO.8352027)

[81] Kubatko LS, Degnan JH. 2007 Inconsistency of phylogenetic estimates from concatenated data under coalescence. Syst. Biol. **56**, 17-24. (10.1080/10635150601146041) 17366134

[82] Freer JJ, Collins RA, Tarling GA, Collins MA, Partridge JC, Genner MJ. 2022 Global phylogeography of hyperdiverse lanternfishes indicates sympatric speciation in the deep sea. Glob. Ecol. Biogeogr. **31**, 2353-2367. (10.1111/geb.13586)

[83] Hodge JR, Read CI, Bellwood DR, van Herwerden L. 2013 Evolution of sympatric species: a case study of the coral reef fish genus *Pomacanthus* (Pomacanthidae). J. Biogeogr. **40**, 1676-1687. (10.1111/jbi.12124)

[84] Rocha LA, Lindeman KC, Rocha CR, Lessios HA. 2008 Historical biogeography and speciation in the reef fish genus *Haemulon* (Teleostei: Haemulidae). Mol. Phylogenet. Evol. **48**, 918-928. (10.1016/j.ympev.2008.05.024) 18599320

[85] Choat JH, Klanten OS, Van Herwerden L, Robertson DR, Clements KD. 2012 Patterns and processes in the evolutionary history of parrotfishes (Family Labridae). Biol. J. Linnean Soc. **107**, 529-557. (10.1111/j.1095-8312.2012.01959.x)

[86] Wainwright PC, Santini F, Bellwood DR, Robertson DR, Rocha LA, Alfaro ME. 2018 Phylogenetics and geography of speciation in New World *Halichoeres* wrasses. Mol. Phylogenet. Evol. **121**, 35-45. (10.1016/j.ympev.2017.12.028) 29289544

[87] Tariel J, Longo GC, Bernardi G. 2016 Tempo and mode of speciation in *Holacanthus* angelfishes based on RADseq markers. Mol. Phylogenet. Evol. **98**, 84-88. (10.1016/j.ympev.2016.01.010) 26876637

[88] Turelli M, Barton NH, Coyne JA. 2001 Theory of speciation. Trends Ecol. Evol. **16**, 330-343. (10.1016/S0169-5347(01)02177-2) 11403865

[89] Coyne JA, Orr HA. 2004 Speciation. Sunderland, MA: Sinauer.

[90] Coyne JA, Orr HA. 1989 Patterns of speciation in Drosophila. Evolution **43**, 362-381. (10.1111/j.1558-5646.1989.tb04233.x) 28568554

[91] Martin PR, Montgomerie R, Lougheed SC. 2010 Rapid sympatry explains greater color pattern divergence in high latitude birds. Evolution **64**, 336-347. (10.1111/j.1558-5646.2009.00831.x) 19744123

[92] Andersen MJ, Shult HT, Cibois A, Thibault J-C, Filardi CE, Moyle RG. 2015 Rapid diversification and secondary sympatry in Australo-Pacific kingfishers (Aves: Alcedinidae: *Todiramphus*). R. Soc. Open Sci. **2**, 140375. (10.1098/rsos.140375) 26064600 PMC4448819

[93] Pigot AL, Tobias JA. 2015 Dispersal and the transition to sympatry in vertebrates. Proc. R. Soc. B **282**, 20141929. (10.1098/rspb.2014.1929) PMC4286046 25621326

[94] Romanuk TN, Hayward A, Hutchings JA. 2011 Trophic level scales positively with body size in fishes. Glob. Ecol. Biogeogr. **20**, 231-240. (10.1111/j.1466-8238.2010.00579.x)

[95] Ingram T. 2011 Speciation along a depth gradient in a marine adaptive radiation. Proc. R. Soc. B **278**, 613-618. (10.1098/rspb.2010.1127) PMC3025674 20810434

[96] Platell ME, Potter IC. 2001 Partitioning of food resources amongst 18 abundant benthic carnivorous fish species in marine waters on the lower west coast of Australia. J. Exp. Mar. Biol. Ecol. **261**, 31-54. (10.1016/S0022-0981(01)00257-X) 11438104

[97] Pfennig DW, Pfennig KS. 2010 Character displacement and the origins of diversity. Am. Nat. **176**, S26-S44. (10.1086/657056) 21043778 PMC3285564

[98] Pigot AL, Tobias JA. 2013 Species interactions constrain geographic range expansion over evolutionary time. Ecol. Lett. **16**, 330-338. (10.1111/ele.12043) 23231353

[99] Brown WL, Wilson EO. 2006 Character displacement. Syst. Zool. **5**, 49. (10.2307/2411924)

[100] Schluter D, McPhail JD. 1992 Ecological character displacement and speciation in sticklebacks. Am. Nat. **140**, 85-108. (10.1086/285404) 19426066

[101] Smith KF, Brown JH. 2002 Patterns of diversity, depth range and body size among pelagic fishes along a gradient of depth. Glob. Ecol. Biogeogr. **11**, 313-322.

[102] Hemingson CR, Cowman PF, Hodge JR, Bellwood DR. 2019 Colour pattern divergence in reef fish species is rapid and driven by both range overlap and symmetry. Ecol. Lett. **22**, 190-199. (10.1111/ele.13180) 30467938

[103] Price SA, Tavera JJ, Near TJ, Wainwright PC. 2013 Elevated rates of morphological and functional diversification in reef-dwelling Haemulid fishes. Evolution **67**, 417-428. (10.1111/j.1558-5646.2012.01773.x) 23356614

[104] Nursall JR. 1974 Character displacement and fish behavior, especially in coral reef communities. Integr. Comp. Biol. **14**, 1099-1118. (10.1093/icb/14.4.1099)

[105] Price SA, Claverie T, Near TJ, Wainwright PC. 2015 Phylogenetic insights into the history and diversification of fishes on reefs. Coral Reefs **34**, 997-1009. (10.1007/s00338-015-1326-7)

[106] Felsenstein J. 1985 Phylogenies and the comparative method. Am. Soc. Natural. **125**, 115. (10.1016/j.jeconom.2010.07.008)

[107] Glass JR, Harrington RC, Cowman PF, Faircloth BC, Near TJ. 2023 Data from: Widespread sympatry in a species-rich clade of marine fishes (Carangoidei). Dryad Digital Repository. (10.5061/dryad.37pvmcvm1) PMC10618865 37909084

[108] Glass JR, Harrington RC, Cowman PF, Faircloth BC, Near TJ. 2023 Widespread sympatry in a species-rich clade of marine fishes (Carangoidei). Figshare. (10.6084/m9.figshare.c.6881855) PMC10618865 37909084

